# Facile synthesis of amorphous coordinated metal–drug frameworks for cancer immunotherapy

**DOI:** 10.1016/j.mtbio.2026.103545

**Published:** 2026-08-10

**Authors:** Ruoyu Cheng, Zehua Liu, Gang Zhao, Han Gao, Wei Huang, Baoding Zhang, Zheng Wang, Jiachen Li, Junyuan Xiao, Yuting Fan, Fuhua Zhang, Jouni Hirvonen, Xianming Deng, Hélder A. Santos, Wenguo Cui

**Affiliations:** a Department of Orthopaedics, Shanghai Key Laboratory for Prevention and Treatment of Bone and Joint Diseases, Shanghai Institute of Traumatology and Orthopaedics, Ruijin Hospital, Shanghai Jiao Tong University School of Medicine, 197 Ruijin 2nd Road, Shanghai, 200025, China; b Department of Biomaterials and Biomedical Technology, The Personalized Medicine Research Institute (PRECISION), University Medical Center Groningen, University of Groningen, AV Groningen, 9713, The Netherlands; c Drug Research Program, Division of Pharmaceutical Chemistry and Technology, Faculty of Pharmacy, University of Helsinki, Helsinki, FI-00014, Finland; d State Key Laboratory of Cellular Stress Biology, School of Life Sciences, Faculty of Medicine and Life Sciences, Xiamen University, 361102, China; e State-Province Joint Engineering Laboratory of Targeted Drugs from Natural Products, School of Life Sciences, Xiamen University, 361102, China; f Department of Integrative Oncology, Fudan University Shanghai Cancer Center, Shanghai, 200032, China; g Department of Pharmacology, School of Medicine, Southeast University, 87 Dingjiaqiao Road, Nanjing, Jiangsu, 210009, China

**Keywords:** Tumor microenvironment, Macrophages, PIKfyve inhibitors, Nanomedicine

## Abstract

Phosphoinositide kinase containing FYVE-type zinc finger (PIKfyve) inhibitors show potential immunomodulatory activity but suffer from poor tumor targeting and formulation challenges. Herein, we screened a series of PIKfyve inhibitors and engineered selected PIKfyve inhibitor into amorphous metal-drug frameworks (aMDFs) through coordination chemistry between pyridine/imidazole moieties and tailored metal nodes verified by theoretical simulations and nuclear magnetic resonance spectroscopy. The resulting aMDFs showed enhanced stability and processability, enabling efficient encapsulation into solid lipid NPs (HF@SLN). In both orthotopic and subcutaneous murine pancreatic cancer models, HF@SLN demonstrated superior tumor growth inhibition over free PIKfyve inhibitors. HF@SLN treatment was associated with increased transcription factor EB (TFEB) expression, accompanied by enhanced cytotoxic T cell infiltration and improved antitumor immune responses in both murine and human pancreatic tumor samples. This study demonstrates the feasibility of employing carrier-free aMDFs for the formulation and delivery of pyridine- and imidazole-containing therapeutics.

## Graphical abstract

A mechanism-guided strategy for constructing amorphous coordinated metal–drug frameworks (aMDFs) for cancer immunotherapy. HW131 was screened as a model PIKfyve inhibitor and directly coordinated with Zn^2+^ through pyridine-, imine-, and carbonyl-mediated multidentate interactions to form carrier-free amorphous nanostructures. The resulting nanoparticles can active the transcription factor EB (TFEB) and polarize the M2 to M1 macrophages, increasing the ratio between CD8^+^ and CD4^+^ T cells.

**Figure:**
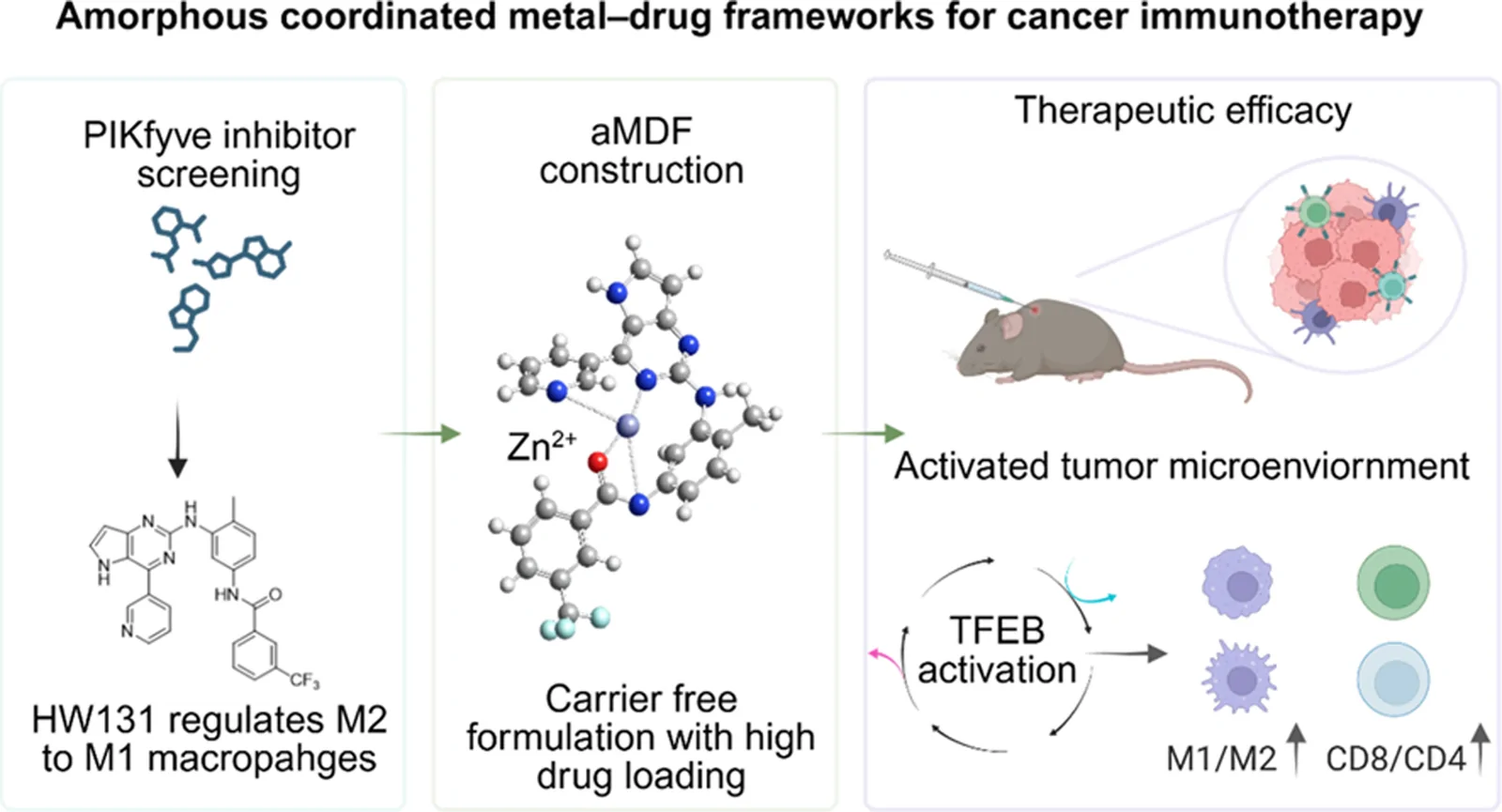

The advent of cancer immunotherapy has promoted substantial advances in expanding the landscape for oncology drugs [1]. In addition to antibody-based blockade of programmed death-1 (PD-1) and programmed death-ligand 1 (PD-L1), increasing attention has been directed toward small-molecule agents capable of modulating antitumor immune responses [2]. Comparing to antibody-based therapeutics, small molecules show advantages in convenient clinical practice, feasible dosage and bioavailability tuning, and more importantly, expanded target space to generate immunostimulatory effects through targeting different signaling pathways with the approval of an increasing number of new drugs or combination regimens [3]. Nevertheless, small molecules have limitation in off-target effects and unsatisfied tumor accumulation, necessitating further formulation design and optimization [4].

In recent years, adoption of metal-organic frameworks nanoparticles (MOFs) as drug delivery vehicles has emerged as an appealing approach due to their high porosity, enzymatic-mimicry property, and biocompatibility [5,6]. Drug-loaded MOFs can be feasibly surface-modified to enhance its *in vivo* performance, enabling targeted or responsive delivery with improved tumor accumulation [7]. Moreover, compared to direct encapsulation of free molecules, drug-loaded MOFs also show enhanced processability for subsequent surface functionalization or encapsulation in a template-directive manner [8]. However, the preparation of some crystalline MOFs requires stringent reaction conditions, prolonged synthesis procedures, or organic solvents that may complicate purification, scale-up, and subsequent biomedical application [9]. For biomedical use, coordination materials that can be prepared under mild and simplified conditions are therefore particularly desirable. Moreover, conventional MOFs are commonly synthesized by the coordination between metal ions (e.g., zinc ions, iron ions, and copper ions) as nodes and bio-inactive organic ligands (e.g., pyridine, imidazole, and triazole) as building fragments [10,11]. Directly employing coordination-capable therapeutic molecules as building components may reduce the requirement for additional organic linkers and simplify formulation composition.

Previous studies have employed bioactive molecules, including nucleotides and small-molecule drugs, to construct metal–organic or metal–drug coordination nanostructures [12]. These studies demonstrated the feasibility of carrier free, or ligand minimized coordination systems, although their formation and material properties remained dependent on the coordination motifs, molecular geometry, and metal binding characteristics of the selected therapeutic molecules. The successful formation of such materials is influenced by several molecular parameters, including the type, number, accessibility, and spatial arrangement of metal-coordinating groups within the therapeutic molecule. In contrast, amorphous metal–organic frameworks (aMOFs) represent a class of noncrystalline coordination materials that retain short range metal–ligand interactions without the long-range order characteristic of crystalline MOFs [13]. Their reduced requirement for long-range structural order may permit greater flexibility in the selection of coordination-capable building components and may facilitate preparation under comparatively mild conditions [14]. This structural flexibility provides a rationale for evaluating whether small molecule therapeutics containing accessible nitrogen donor groups can be assembled into amorphous aMDFs with improved formulation properties.

In this study, we investigated the feasibility of constructing amorphous molecular drug frameworks (aMDFs) from coordination-capable phosphoinositide kinase containing FYVE-type zinc finger (PIKfyve) inhibitors. A series of PIKfyve inhibitors were screened through computational analysis and formulation evaluation to identify molecules compatible with coordination-driven self-assembly. Among the candidates, HW131 was selected based on its favorable coordination behavior and formulation characteristics for the construction of aMDFs. The resulting aMDFs were subsequently encapsulated within solid lipid nanoparticles (HF@SLN) to improve their colloidal stability and biological applicability. We systematically characterized the physicochemical properties of HF@SLN and evaluated its biological activity and therapeutic efficacy in vitro and in two murine pancreatic tumor models. Overall, this work aimed to demonstrate the feasibility of employing an amorphous metal–drug coordination strategy to formulate a PIKfyve inhibitor and highlighting the potential to improve nanoparticle stability, delivery performance, and antitumor immunotherapy.

## 1 Construction and characterization of PIKfyve inhibitor-based aMDFs

**Fig. 1:**
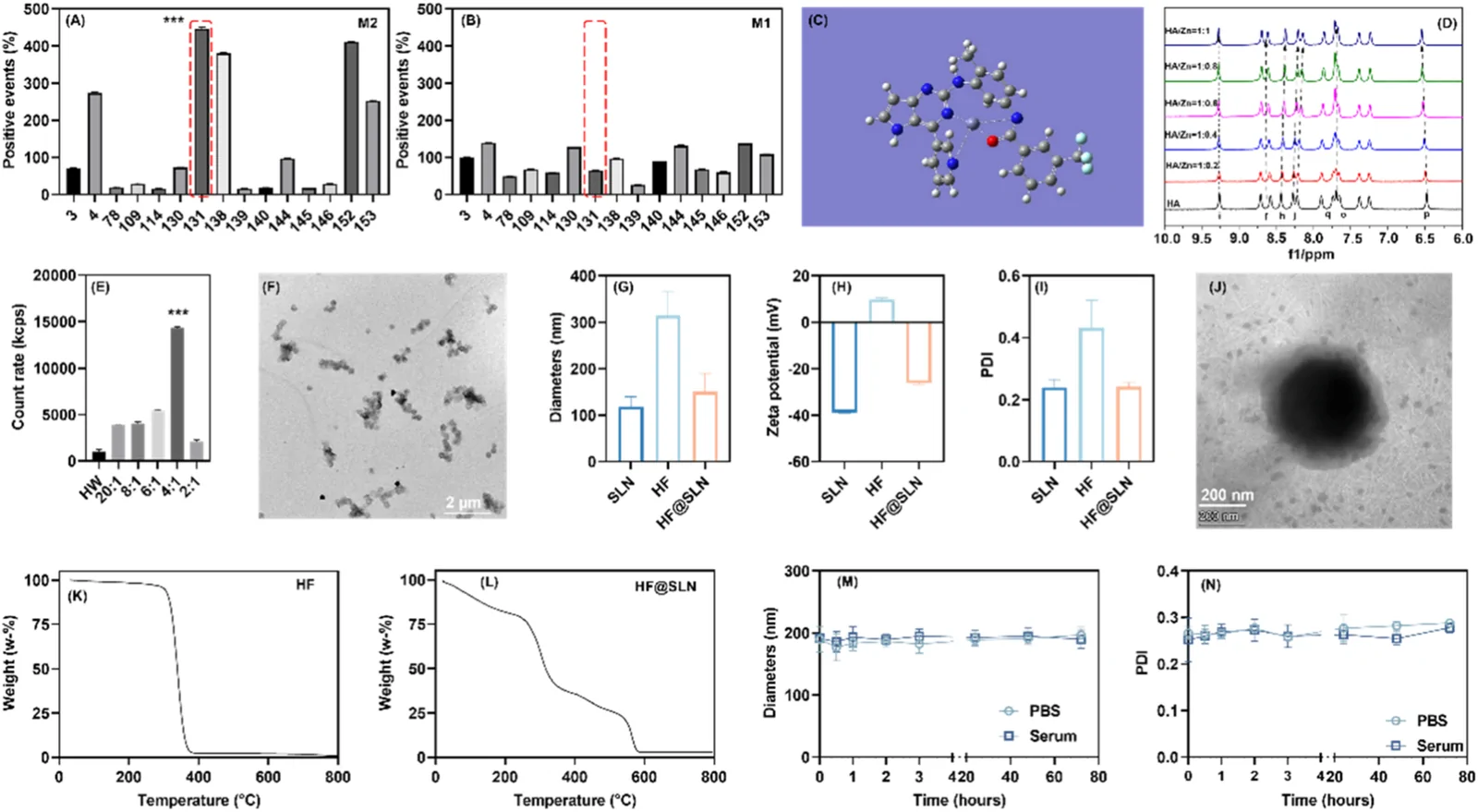
**Preparation of HF@SLN NPs.** Bioactivities of 15 isomers in (**A**) M2 BMDMs and (**B**) M1 BMDMs. (**C**) Theoretical simulation calculation between HW and Zn^2+^, red dots (oxygen), blue dots (nitrogen), light blue dots (fluorine), grey dots (carbon), light grey dots (hydrogen), purple dots (zinc). (**D**) ^1^H NMR titration results of different molar ratio between HW and Zn^2+^. (**E**) Counting rate of HF NPs prepared with different molar ratio between HW and Zn^2+^. (**F**) The TEM images of HF. Evaluating SLN, HF, and HF@SLN in (**G**) diameter, (**H**) zeta-potential, and (**I**) PDI. (**J**) TEM images of HF@SLN. TGA in (**K**) HF and (**L**) HF@SLN. Evaluating the stability of HF@SLN in (**M**) diameter and (**N**) PDI. Data are presented as means ± standard deviation (SD). The error bars are based on standard errors of the individual samples (n = 3), ***P < 0.001, ANOVA with Tukey's post-test. (For interpretation of the references to colour in this figure legend, the reader is referred to the Web version of this article.)

Given that PIKfyve inhibition has recently emerged as a promising strategy to potentiate cancer immunotherapy through macrophage reprogramming [15]. We first sought to identify a potent PIKfyve inhibitor suitable for constructing bioactive aMDFs. Fifteen previously reported PIKfyve inhibitors were synthesized and structurally confirmed by the ^1^H nuclear magnetic resonance (^1^H NMR, Fig. S1). Their autophagy inhibition activities were subsequently evaluated in both M1-and M2-polarized bone marrow-derived macrophages (BMDMs) by assessing cytoplasmic vacuolization, a hallmark of effective PIKfyve inhibition [16]. Qualitative microscopic observation (Fig. S2) together with quantitative flow cytometric analysis of lysosomal biogenesis revealed that HW-05-131-01 (hereafter denoted as HW) induced the strongest vacuolization response in M2 BMDMs while maintaining substantially lower activity in M1 BMDMs (Fig. 1a and b). Specifically, HW increased vacuolization in M2 BMDMs to 446 ± 6%, whereas only a modest enhancement (65 ± 1%) was observed in M1 BMDMs, resulting in the highest M2/M1 vacuolization ratio (6.8) among all tested compounds. These findings indicate that HW possesses both potent autophagy inhibition capability and preferential activity toward immunosuppressive M2 macrophages, making it an ideal candidate for constructing functional aMDFs.

Despite its potent bioactivity, the direct encapsulation efficiency of free HW remained relatively low (26.4 ± 3.8%), which may limit its translational potential. Previous studies have demonstrated that pre-organizing hydrophobic therapeutics into particulate intermediates can markedly improve drug processability and encapsulation efficiency [[17], [18], [19]]. Inspired by these observations, we explored the coordination-driven assembly of HW with metal ions to generate aMDFs. Initially, Cu^2+^ was employed as the coordinating metal ion. However, the resulting nanoparticles exhibited poor colloidal uniformity with a polydispersity index (PDI) exceeding 0.3, indicating heterogeneous particle formation. In contrast, coordination between HW and Zn^2+^ generated nanoparticles with narrower size distributions, suggesting more controllable amorphous assembly behavior. Therefore, Zn^2+^ was selected for subsequent studies.

By the theoretical simulation calculation, the Zn^2+^ can interact with pyridine (N), imine (NH), NH-C=O (N), and C=O (O) with a distance of 3.69 Å, 1.95 Å, 3.10 Å, and 1.85 Å, which can theoretically form the chelates (Fig. 1c). According to the results of ^1^H NMR titration, HW showed chemical shift on 9.26, 8.57, 8.43, 8.27-8.21 ppm, 7.73-7.64 ppm, and 6.48 ppm, belonging to pyridine, imidazole ring, and benzene, respectively. After interacting with Zn^2+^, the peaks of 9.26 ppm (pyridine) and 8.57 ppm (imidazole ring) shifted to the high field. In addition, -NH coordinated with Zn^2+^ causing a shift of 6.48 ppm (benzene) to the high field. With the increasing amount of Zn^2+^, the peak at 8.65 ppm (pyridine and benzene) shifted to the low field; in addition, the peak shape changed from three peaks to two peaks (Fig. 1d). These ^1^H NMR titrations indicated that Zn^2+^ coordinated with N from pyridine, imine and benzene, and NH and C=O from Ar-NH-C=O, which is consistent with the theoretical simulation calculation.

We further investigated the preparation of NPs by using the different molar ratios between HW and Zn^2+^. The synthesis of NPs was confirmed by the counting rate using dynamic light scattering (DLS) measurements. The NPs (consisting of the coordination between HW and Zn^2+^ were denoted as HF in the following content) synthesized with the molar ratio at 4:1 (HW: Zn^2+^) showed the highest counting rate (14319 ± 124 kcps, Fig. 1e), indicating the formation of HF NPs. Furthermore, transmission electron microscopy (TEM) images also proved the successful synthesis of HF (Fig. 1f). TEM images of pure HW are shown in Fig. S3a.

Although metal-coordinated amorphous nanoparticles offer high drug loading capacity, their colloidal instability under physiological conditions remains a major limitation due to competitive interactions with ions and serum proteins [20]. To address this challenge, HF nanoparticles were further coated with a solid lipid nanoshell (SLN) to generate core–shell HF@SLN nanoparticles. This template-directed encapsulation strategy substantially improved nanoparticle stability while maintaining a high encapsulation efficiency of 78.6 ± 6.5% and loading efficiency of 6.7 ± 2.1%. Interestingly, HF@SLN exhibited a reduced hydrodynamic diameter (150 ± 40 nm) compared to bare HF nanoparticles (314 ± 52 nm) (Fig. 1g and h), likely because the SLN shell prevented further uncontrolled coordination-driven aggregation during particle maturation [21,22]. Concurrently, the surface zeta potential shifted from +10.0 ± 0.6 mV for HF to −26.1 ± 0.4 mV for HF@SLN, confirming successful lipid shell coating. Furthermore, HF@SLN displayed a more reduced PDI (0.24 ± 0.01) relative to bare HF nanoparticles (0.43 ± 0.09), indicating enhanced colloidal homogeneity (Fig. 1i), with the yield around 73.2 ± 8.7%.

TEM imaging further revealed a distinct core-shell morphology consisting of an electron-dense HF core surrounded by a lighter lipid shell (Fig. 1j). Thermogravimetric analysis (TGA) additionally confirmed the presence of the SLN coating. Unlike HF nanoparticles, which underwent rapid single-stage degradation between 300 and 400 °C, HF@SLN exhibited characteristic multistage weight loss profiles attributable to sequential lipid decomposition and HF degradation (Fig. 1k and l) [23]. X-ray diffraction (XRD) analysis further demonstrated the amorphous nature of HF@SLN and the successful masking of HF crystalline diffraction peaks by the SLN shell (Fig. S3b–e). Importantly, HF@SLN remained highly stable in both phosphate-buffered saline (PBS) and serum containing medium, maintaining particle diameters below 200 nm and PDIs below 0.3 over time (Fig. 1m and n), highlighting the effectiveness of lipid shell stabilization.

To investigate the generalizability of this coordination-driven strategy, additional therapeutics containing pyridine or imidazole motifs, including sorafenib, pexidartinib, and erlotinib, were coordinated with Zn^2+^. All resulting formulations formed nanoparticles with hydrodynamic sizes of approximately 200 nm, negative surface charges, and narrow PDIs (Fig. S4). These preliminary results suggest that Zn-mediated coordination may be extended to other structurally related small-molecule drugs possessing suitable coordination motifs. However, comprehensive physicochemical characterization and biological evaluation of these formulations were beyond the scope of the present study.

## 2 HF@SLN alters macrophage polarization markers and increases TFEB expression

**Fig. 2:**
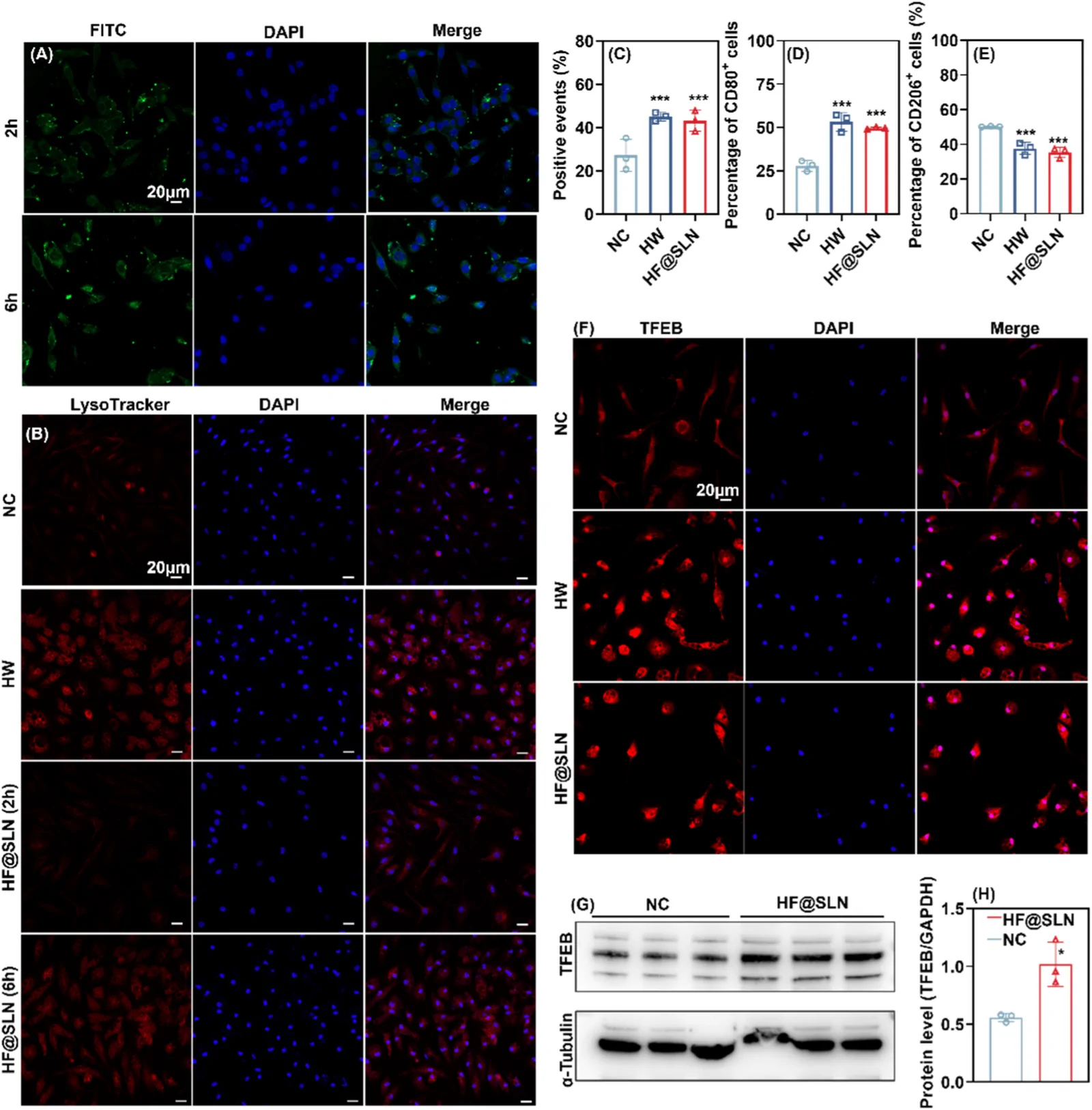
**The interaction between HF@SLN and M2 BMDMs *in vitro*.** (**A**) Confocal images of internalized HF@SLN by the M2 BMDMs, cell nucleus was stained by 4′,6-diamidino-2-phenylindole (DAPI, blue), and HF@SLN was connected with FITC (green); scale bar 20 μm. (**B**) Confocal images of lysosome in M2 BMDMs treated with HW, HF@SLN or without any treatments (denoted as NC), cell nucleus was stained by DAPI (blue), and lysosome was stained with Lysotracker (red); scale bar 20 μm. (**C**) Quantitative analysis of lysosome. Quantitative analysis of (**D**) CD80^+^ and (**E**) CD206^+^ macrophages. (**F**) Confocal images of TFEB expression on the M2 BMDMs treated with HW, HF@SLN or without any treatments (denoted as NC), cell nucleus was stained by DAPI (blue) and TFEB was stained with immunofluorescence staining (red); scale bar 20 μm. (**G**) Western blotting images of TFEB expression on the M2 BMDMs treated with HF@SLN or without any treatment (denoted as NC). (**H**) The quantitative results of Western blotting images. Data are presented as means ± SD. The error bars are based on standard errors of the individual samples (n = 3), *P < 0.05 and ***P < 0.001, ANOVA with Tukey's post-test. (For interpretation of the references to colour in this figure legend, the reader is referred to the Web version of this article.)

Following successful nanoparticle fabrication, we next investigated the biological interactions of HF@SLN with macrophages in vitro. Fluorescein isothiocyanate (FITC)-labeled HF@SLN nanoparticles exhibited time-dependent association with M2 BMDMs, with substantially stronger cellular fluorescence observed after 6 h compared to 2 h incubation (Fig. 2a), indicating progressive nanoparticle internalization.

Given that PIKfyve inhibition disrupts lysosomal maturation and induces cytoplasmic vacuolization, we next evaluated lysosomal alterations using LysoTracker staining. Untreated M2 BMDMs displayed relatively weak lysosomal fluorescence, whereas both free HW and HF@SLN treatments induced markedly enhanced lysosomal accumulation after 6 h incubation (Fig. 2b). Quantitative flow cytometric analysis further confirmed a more elevated lysosomal signals in both treatment groups, with HF@SLN and free HW inducing 1.67 ± 0.52 fold and 1.74 ± 0.49 fold increases, respectively, relative to untreated controls (Fig. 2c). Notably, no statistically significant difference was observed between free HW and HF@SLN, indicating that nanoparticle assembly preserved the intrinsic PIKfyve inhibitory activity of HW.

Accumulating evidence suggests that lysosomal stress and autophagy disruption regulate multiple aspects of macrophage function, including changes in polarization-associated phenotypes [24,25]. Given the established role of PIKfyve inhibition in regulating macrophage function, we next evaluated the effects of HF@SLN treatment on macrophage polarization markers in M2-polarized BMDMs. Flow cytometric analysis revealed marked changes in macrophage polarization marker expression following treatment with either free HW or HF@SLN (Fig. 2d and e). Specifically, the proportion of CD80-positive macrophages increased from 27.9 ± 3.1% in untreated controls to 53.2 ± 5.3% and 49.6 ± 0.7% after HW and HF@SLN treatment, respectively, whereas the proportion of CD206-positive macrophages was concomitantly reduced. These findings indicate that HF@SLN treatment is associated with changes in macrophage polarization marker expression consistent with a pro-inflammatory phenotype.

Previous studies have linked PIKfyve inhibition to activation of TFEB, a master regulator of lysosomal biogenesis and macrophage functional reprogramming [[26], [27], [28]]. Consistent with these reports, immunofluorescence staining revealed markedly elevated TFEB expression following HW and HF@SLN treatment (Fig. 2f). Western blot analysis further demonstrated that HF@SLN increased TFEB expression by approximately 1.8 ± 0.5-fold compared to untreated controls (Fig. 2g and h). The increased TFEB expression was accompanied by macrophage repolarization toward a pro-inflammatory phenotype, suggesting a potential involvement of TFEB in the immunomodulatory effects of HF@SLN.

## 3 HF@SLN remodels the tumor immune microenvironment and suppresses pancreatic tumor progression

**Fig. 3:**
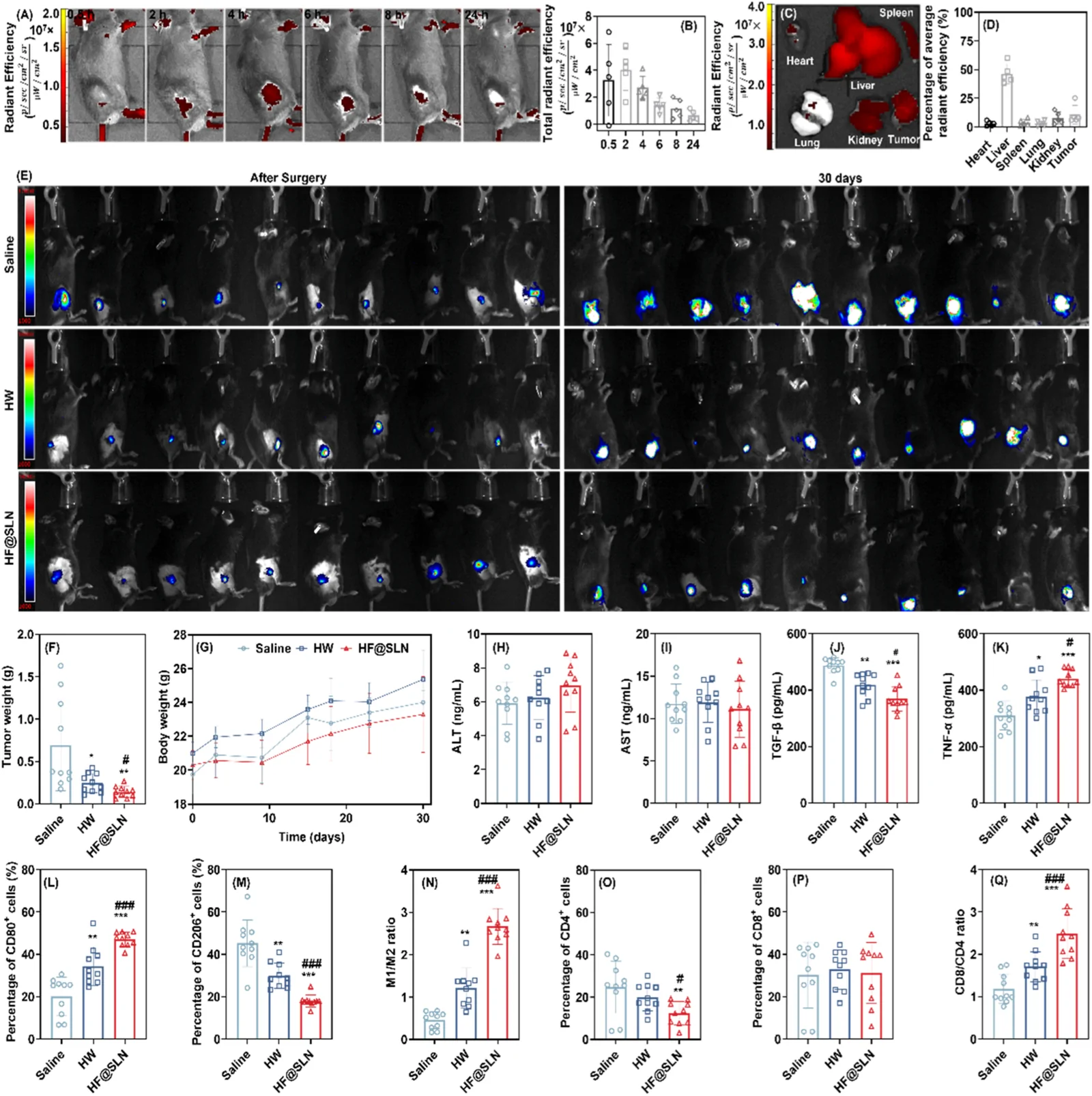
**Biodistribution, biosafety, and therapeutic effects of HF@SLN NPs in the subcutaneous pancreatic cancer model.** (**A**) Biodistribution of cyanine 7-labeled HF@SLN and (**B**) quantitative analysis in tumor tissues (n = 5), after intravenous injection. (**C**) Biodistribution of cyanine 7-labeled HF@SLN and (**D**) quantitative analysis in main organs (n = 5), after 4 h intravenous injection. (**E**) IVIS images of mice from all groups after surgery and on day 30 (n = 10). (**F**) Tumor weight of each mouse (n = 10). (**G**) Body weight of mice (n = 10). Serum concentration of (**H**) ALT and (**I**) AST in mice (n = 10). Serum concentration of (**J**) TGF-β and (**K**) TNF-α in mice (n = 10). The percentages of (**L**) CD80^+^ macrophages, (**M**) CD206^+^ macrophages, and (**N**) the ratio of CD80^+^/CD206^+^ macrophages in mice (n = 10). The percentages of (**O**) CD4^+^ T cells, (**P**) CD8^+^ T cells, and (**Q**) the ratio of CD8^+^/CD4^+^ T cells in mice (n = 10). Data are presented as means ± SD. The error bars are based on standard errors of the individual animals. *Indicates the statistically significant differences comparing HW and HF@SLN with saline. ^#^Statistically significant differences of HW and HF@SLN. ***P < 0.001, **P < 0.01, *P < 0.05 *^, ###^P < 0.001, and ^#^P < 0.05. ANOVA with Tukey's post-test.

We further explored whether the HF@SLN can reprogram M2 BMDMs *in vivo*. First, we investigated the biodistribution of HF@SLN by intravenously injecting cyanine 7-labeled HF@SLN into subcutaneous Pan02-bearing mice. The accumulation of HF@SLN reached the peak value in the tumor 4 h after injection; in addition, there was no apparent HF@SLN accumulation in the tumor 24 h after injection, which is proved by both qualitative and quantitative analysis (Fig. 3a and b). Moreover, 4 h after injection, most HF@SLN located in the liver (46.4 ± 8.6%), followed by the tumor (10.8 ± 8.1%), kidney (7.9 ± 5.1%), spleen (3.9 ± 2.9%), lung (3.2 ± 2.2%), and heart (2.9 ± 2.0%) (Fig. 3c and d), which was consistent with the expected biodistribution behavior of lipid-based nanomaterials [29].

The therapeutic efficacy of HF@SLN was first evaluated in a postoperative recurrence model of subcutaneous pancreatic cancer. Partial tumor resection was performed to mimic clinically relevant residual disease. Two days after surgery, tumors were visualized, and the mice were randomly divided into three groups with different treatments (Saline, HW+50 mg/kg gemcitabine, and HF@SLN+50 mg/kg gemcitabine). After 30 days of treatments, the tumors were visualized again, and inhibited tumor growth was observed in both HW and HF@SLN groups (Fig. 3e). In addition, the HF@SLN groups showed no statistically significant difference in tumor weight (0.14 ± 0.07g) compared to the HW groups (0.25 ± 0.11g) and saline groups (0.69 ± 0.54g) (Fig. 3f). During the whole therapeutic process, the average body weight steadily increased in all groups without statistically significant differences (Fig. 3g). Additionally, the histology of main organs, such as the heart, liver, spleen, lung, and kidney, was organized in all groups (Fig. S5). The concentration of alanine transaminase (ALT) and aspartate transaminase (AST) were similar among the groups without statistically significant differences (Fig. 3h and i), indicating that liver function was normal in all groups. Increasing body weights, organized histology of main organs, and normal liver functions proved the biosafety of HW and HF@SLN during the therapeutic process.

We next examined whether HF@SLN-mediated tumor suppression was associated with remodeling of the tumor immune microenvironment (TME). Cytokine analysis revealed that HF@SLN statistically significantly reduced the immunosuppressive cytokine (transforming growth factor beta, TGF-β, 368.6 ± 43.9 pg/ml) while simultaneously increasing pro-inflammatory (tumor necrosis factor-alpha, TNF-α, 441.1 ± 30.4 pg/mL) levels compared to the HW (TGF-β: 418.2 ± 47.8 pg/ml and TNF-α: 376.5 ± 59.7 pg/mL) and saline (TGF-β: 487.4 ± 27.4 pg/mL and TNF-α: 309.9 ± 50.9 pg/mL) groups (Fig. 3j and k), suggesting conversion of the TME from an immune-suppressive to immune-activated state.

Flow cytometric analysis further demonstrated pronounced macrophage repolarization within tumors following HF@SLN treatment. Specifically, tumors from each mouse were prepared for sing-cells suspensions for the following flow cytometry analysis (gating strategy was presented in Fig. S6–9). Mice treated with HF@SLN, and HW, respectively, exhibited increased percentages of CD80^+^ macrophages (HF@SLN: 47.1 ± 3.3%, HW:34.5 ± 9.1%, and Saline:20.3 ± 9.0%) and decreased percentages of CD206^+^ macrophages (HF@SLN: 18.0 ± 2.8%, HW:30.0 ± 5.9%, and saline: 45.3 ± 11.0%) compared to the saline groups (Fig. 3l–n). Additionally, there was a decreased percentage of CD4^+^ T cells in mice treated with HF@SLN (Fig. 3o), but there were no statistically significant differences in CD8^+^ T cells in all groups (Fig. 3p). However, mice treated with HF@SLN and HW presented a higher CD8^+^/CD4^+^ ratio than the Saline group (HF@SLN: 2.5 ± 0.6, HW:1.7 ± 0.4, and saline: 1.2 ± 0.4, (Fig. 3q), indicating a more cytotoxic immune landscape.

**Fig. 4:**
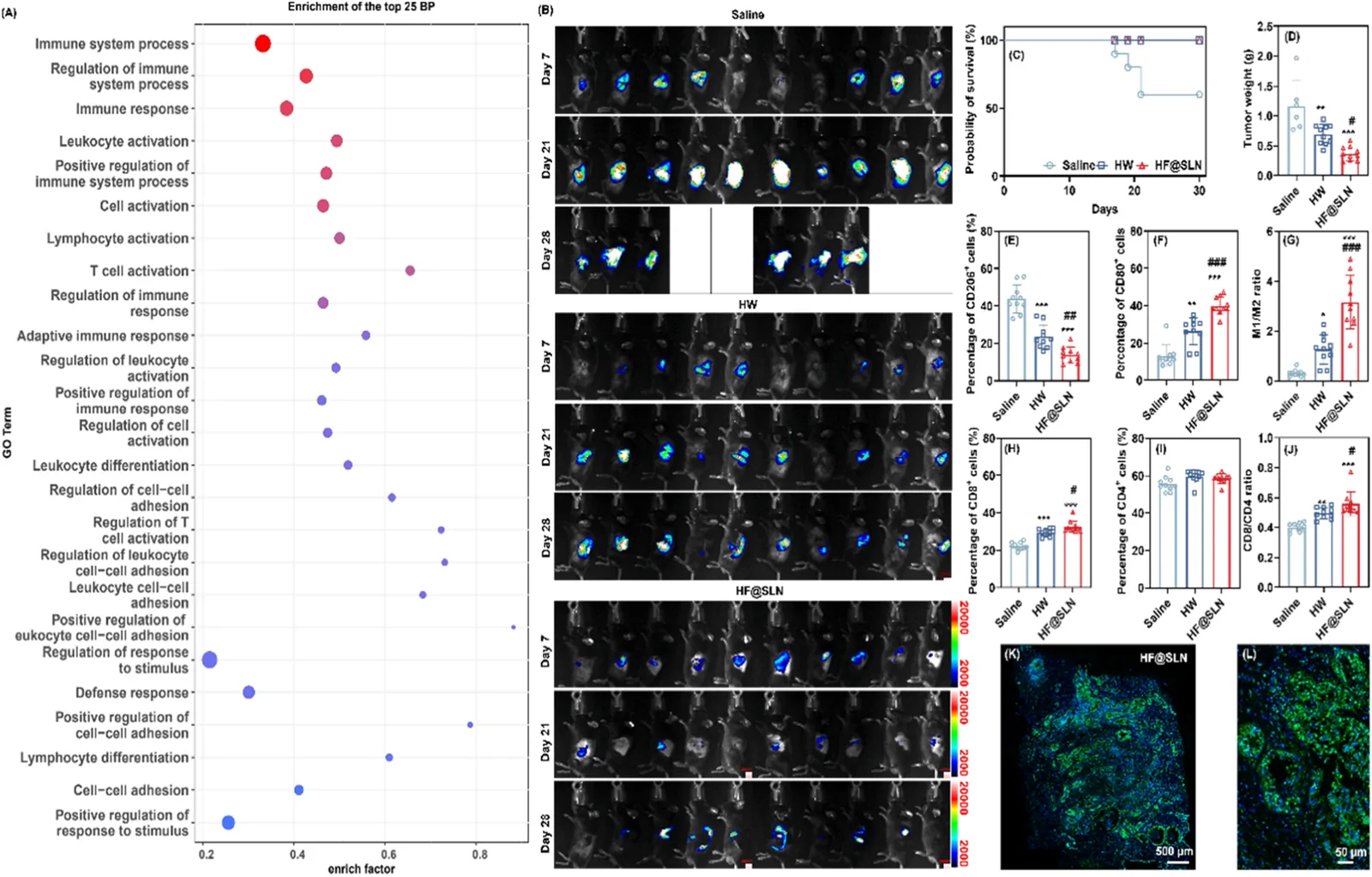
**The therapeutic effects and potent immunotherapeutic machines of HF@SLN NPs in the orthotopic pancreatic cancer model.** (**A**) The top 25 enhanced biological processes on mice treated with HF@SLN compared to the saline in the transcriptome analysis. (**B**) IVIS images of mice from all groups on day 7, 21, and 30 (n = 10). (**C**) Survival rate of Pan02 orthotopic tumor-bearing mice treated with saline, HW, and HF@SLN (n = 10). (**D**) Tumor weight of mice treated with saline (n = 6), HW and HF@SLN (n = 10). Percentages of (**E**) CD206^+^ macrophages, (**F**) CD80^+^ macrophages, and (**G**) the ratio of CD80^+^/CD206^+^ macrophages in mice (n = 10). Percentages of (**H**) CD8^+^ T cells, (**I**) CD4^+^ T cells, and (**J**) the ratio of CD8^+^/CD4^+^ T cells in mice (n = 10). Immunofluorescence staining results of (**K**) human tumour treated HF@SLN in the expression of TFEB and (**L**) the local magnification. Data are presented as means ± SD. The error bars are based on standard errors of the individual animals. *Indicates the statistically significant differences comparing HW and HF@SLN with Saline. ^#^Statistically significant differences of HW and HF@SLN. ***P < 0.001, **P < 0.01, ^###^P < 0.001, ^##^P < 0.01, and ^#^P < 0.05. ANOVA with Tukey's post-test.

To further elucidate the molecular basis underlying HF@SLN-mediated immune remodeling, transcriptomic analysis was performed on tumors collected from saline- and HF@SLN-treated mice. Gene ontology enrichment analysis revealed substantial activation of immune-associated biological processes following HF@SLN treatment, including leukocyte activation, lymphocyte activation, T-cell activation, adaptive immune responses, and immune system processes (Fig. 4a). These transcriptomic findings strongly support the ability of HF@SLN to convert immunologically cold pancreatic tumors into more immune-responsive microenvironments.

Encouraged by these results, we further evaluated HF@SLN in an orthotopic pancreatic tumor model that more faithfully recapitulates clinical pancreatic cancer progression. As shown in Fig. 4b, most mice developed orthotopic pancreatic cancer after seven days. Then the mice were respectively treated with saline, HW+50 mg/kg gemcitabine, and HF@SLN+50 mg/kg gemcitabine, and the tumor situation was continuously recorded after 21 and 28 days. All mice treated with HW or HF@SLN survived throughout the treatment period, whereas four mice in the saline group died during therapy (Fig. 4c). The gradually increased body weight was observed in all groups (Fig. S10). After 30 days of treatments, HF@SLN treatment also achieved the strongest tumor growth inhibition, reducing tumor weight to 0.37 ± 0.12 g compared to saline group (1.16 ± 0.44g) and HW group (0.69 ± 0.17g) (Fig. 4d).

Consistent with observations in the subcutaneous model, HF@SLN profoundly remodeled the orthotopic pancreatic TME by decreasing CD206^+^ macrophages, increasing CD80^+^ macrophages, and enhancing CD8^+^ T cell infiltration. Notably, the HF@SLN group exhibited the highest CD8^+^/CD4^+^ ratio together with the lowest M2/M1 macrophage ratio, further supporting effective immune activation within tumors. Specifically, after processing the tumor tissues into the single cell suspension, the saline group had the highest percentage of CD206^+^ macrophages (43.78 ± 7.47%) compared to the HW group (23.56 ± 6.35%) and HF@SLN (13.87 ± 4.15%) (Fig. 4e). The lowest percentage of CD80^+^ macrophages (13.05 ± 5.97%) was observed in the saline group compared to the HW (26.39 ± 7.21%) and HF@SLN (39.91 ± 4.63%) groups (Fig. 4f). Interestingly, the HF@SLN group presented the highest CD8^+^ percentage of T cells (32.43 ± 3.17%) followed by the HW (29.50 ± 1.88%) and saline (22.23 ± 1.86%) groups. However, there were no statistically significant differences in all groups CD4^+^ percentage of T cells (Fig. 4h and i). HW and HF@SLN groups exhibited an increased M2/M1 ratio and CD8^+^/CD4^+^ compared to the saline groups, indicating the activated TME (Fig. 4g–j). Finally, ex vivo treatment of human pancreatic tumor tissues with HF@SLN induced robust TFEB activation compared with free HW or saline controls (Fig. 4k, l and Fig. S11), highlighting the translational potential of this strategy in clinically relevant human tumor tissues.

Overall, these findings demonstrate that coordination-driven assembly of bioactive therapeutics into aMDFs represents a versatile strategy for simultaneously improving drug delivery and preserving intrinsic pharmacological activity. By integrating PIKfyve inhibition with nanostructure-mediated tumor delivery, HF@SLN effectively activates TFEB signaling, reprograms immunosuppressive macrophages, remodels the pancreatic tumor microenvironment, and potentiates antitumor immunity, thereby providing a promising nanomedicine platform for enhancing pancreatic cancer immunotherapy.

## CRediT authorship contribution statement

**Ruoyu Cheng:** Investigation, Methodology, Project administration, Writing – original draft, Writing – review & editing. **Zehua Liu:** Funding acquisition, Investigation, Methodology, Project administration, Resources, Supervision, Writing – original draft, Writing – review & editing. **Gang Zhao:** Investigation, Methodology, Writing – review & editing. **Han Gao:** Investigation, Methodology, Writing – review & editing. **Wei Huang:** Investigation, Methodology, Writing – review & editing. **Baoding Zhang:** Investigation, Methodology, Writing – review & editing. **Zheng Wang:** Investigation, Methodology, Writing – review & editing. **Jiachen Li:** Investigation, Methodology, Writing – review & editing. **Junyuan Xiao:** Investigation, Methodology, Writing – review & editing. **Yuting Fan:** Writing – review & editing. **Fuhua Zhang:** Writing – review & editing. **Jouni Hirvonen:** Supervision, Writing – review & editing. **Xianming Deng:** Funding acquisition, Project administration, Resources, Supervision, Writing – review & editing. **Hélder A. Santos:** Funding acquisition, Project administration, Resources, Supervision, Writing – review & editing. **Wenguo Cui:** Funding acquisition, Project administration, Resources, Supervision, Writing – review & editing.

## Declaration of competing interests

The authors declare that they have no known competing financial interests or personal relationships that could have appeared to influence the work reported in this paper.

## Data Availability

Data will be made available on request.
