# Supplementary material for: Facile synthesis of amorphous coordinated metal–drug frameworks for cancer immunotherapy
Source: Mater Today Bio. 2026 Aug 10;40:103545. doi: 10.1016/j.mtbio.2026.103545 (PMC13499143; doi:10.1016/j.mtbio.2026.103545)
Supplement: Supplementary file 1 [file mmc1.docx]

Supplementary Materials for

**Facile synthesis of amorphous coordinated metal–drug frameworks for cancer immunotherapy**

Ruoyu Cheng^1,2,3,†^, Zehua Liu^3, †^, Gang Zhao^1^, Han Gao^2,7^, Wei Huang^4, 5^, Baoding Zhang^4, 5^, Zheng Wang^4, 5^, Jiachen Li^2^, Junyuan Xiao^6^, Yuting Fan^3^, Fuhua Zhang^3^, Jouni Hirvonen^3^, Xianming Deng^4,5, *^, Hélder A. Santos^1,2,*^, Wenguo Cui^1,*^

1. R. Cheng, G. Zhao, W. Cui, H. A. Santos

Department of Orthopaedics, Shanghai Key Laboratory for Prevention and Treatment of Bone and Joint Diseases, Shanghai Institute of Traumatology and Orthopaedics, Ruijin Hospital, Shanghai Jiao Tong University School of Medicine, 197 Ruijin 2nd Road, Shanghai 200025, P. R. China.

E-mail: [wgcui@sjtu.edu.cn](mailto:wgcui@sjtu.edu.cn)

2. H. Gao, R. Cheng, J. Li, H. A. Santos

Department of Biomaterials and Biomedical Technology, The Personalized Medicine Research Institute (PRECISION), University Medical Center Groningen, University of Groningen, AV Groningen, 9713 The Netherlands

E-mail: [h.a.santos@umcg.nl](mailto:h.a.santos@umcg.nl)

3. R. Cheng, Z. Liu, Yuting Fan, Fuhua Zhang, J. Hirvonen

Drug Research Program, Division of Pharmaceutical Chemistry and Technology, Faculty of Pharmacy, University of Helsinki, FI-00014 Helsinki, Finland

4. W. Huang, B. Zhang, Z. Wang, X. Deng

State Key Laboratory of Cellular Stress Biology, School of Life Sciences, Faculty of Medicine and Life Sciences, Xiamen University, 361102, China

E-mail: [xmdeng@xmu.edu.cn](mailto:xmdeng@xmu.edu.cn)
5. W. Huang, B. Zhang, X. Deng

State-Province Joint Engineering Laboratory of Targeted Drugs from Natural Products, School of Life Sciences, Xiamen University, 361102, China

6. J. Xiao

Department of Integrative Oncology, Fudan University Shanghai Cancer Center, 200032 Shanghai, China

7. H. Gao

Department of Pharmacology, School of Medicine, Southeast University, 87 Dingjiaqiao Road, Nanjing 210009, Jiangsu, China

^†^ These authors contributed equally to this work.

**EXPERIMENTAL METHODS**

**Cell culture**

Pan02 and Pan02-Luc cells were cultured in Dulbecco’s Modified Eagle’s Medium (DMEM)-high glucose (HyClone) supplemented with 10% fetal bovine serum (FBS, Gibico, 16000-044), and 1% penicillin-streptomycin (HyClone). The cell incubator (Heracell™ VIOS 160i CO_2_ Incubator, Thermo Fisher) was set at 37°C, 5% CO_2_, and 95% relative humidity. The cell culture medium was refreshed every two days, and cells were passaged when the cell density arrived at around 80%.

Bone marrows were isolated from the tibia and fibula of male C57BL/6J mice (6 weeks), after lysis of red blood cells, these bone marrow monocytes were seeded at culture dishes (3×10^5^ cell/ml) in the Roswell’s Park Memorial Institute (RPMI)-1640 (HyClone) supplemented with 10% FBS, 1% penicillin-streptomycin, and 20 ng/ml recombinant human macrophage-stimulating factor (M-CSF, PeproTech, P09603). After 3 days’ incubation, half of the medium was discarded, and the fresh medium was supplemented for the following 3-day culturing. After 6 days’ incubation, the bone marrow derived macrophages (BMDMs) were obtained. BMDMs were cultured with 100 ng/ml lipopolysaccharide (LPS, Sigma-Aldrich, L6529) and 50 ng/ml interferon-γ (IFN-γ, PeproTech, P01580) for 24 h to stimulate the differentiation of M1 BMDMs, with 20 ng/ml interleukin-4 (IL-4) and 20 ng/ml IL-10 for 24 h to stimulate the differentiation of M2 BMDMs.

**Animals**

Male C57BL/6J mice (6-8 weeks) were purchased from Shanghai Jiesijie Laboratory Animal Co., Ltd. C57BL/6J mice and were fed under the protocols approved by Animal Experiment Center, Shanghai Jiaotong University (Number: SYXK2018-0027). The animal experiments were in accordance with international ethics guidelines and the National Institutes of Health Guide, concerning the Care and Use of Laboratory Animals.

**Human samples**

The tumor tissues came from the pancreatic ductal adenocarcinom patients with untreated, resectable, non-metastatic pancreatic tumors. Additionally, these patients were free of infectious diseases, rheumatic diseases, or other tumors. All the human samples were collected from Ruijin Hospital, Shanghai Jiaotong University School of Medicine (Number:2021161). The study protocol was approved by the Ethics Committee of Ruijin Hospital affiliated to School of Medicine, Shanghai Jiaotong University. All enrolled participants consented to attend this cohort study and signed written informed consent. It was not appropriate or possible to involve patients or the public in the design, or conduct, or reporting, or dissemination plans of our research chemical synthesis.

**Investigating the biological activities of chemicals**

Fifteen chemicals were synthesized as described previously ^30^. The M1 and M2 BMDMs were respectively incubated with 10 µM fifteen isomers for 2 h. Then the vacuolization in both M1 and M2 BMDMs were stained by the LysoTracker staining (LysoTracker Red DND-99, Yeasen, 40739ES50) and quantitatively analyzed by the flow cytometry (CytoFLEX S Flow Cytometer, Backman).

**NPs preparation and investigation**

The interaction between HW and Zn^2+^ was explored by the theoretical simulation calculation (Gaussian 03), according to the density functional theory. Further, titration ^1^H-NMR was utilized to evaluate the molar ratios between HW and Zn^2+^. Afterwards, NPs (denoted as HF) were prepared with different mole ratios (20:1, 8:1, 6:1, 4:1, and 2:1) between HW and Zn^2+^ in the methanol at 60℃, then the formation of HF was monitored by the dynamic light scattering (DLS, Malvern Zetasizer) in the counting rate. Furthermore, the cetyl palmitate and 1,2-distearoyl-sn-glycero-3-phosphoethanolamine (mPEG-DSPE) in methanol (mole ratio:100:2) were added into the HF methanol solution at 60℃, followed by adding 2% pluronic-F68 solution to prepared the HF coated with solid lipid nano-shell (denoted as HF@SLN). Then, the HF@SLN was frozen drying and the weight was recorded. The yield of HF@SLN was calculated as the following formula.

$The yield=\frac{m_{HW}+m_{{Zn}^{2+}}+m_{SLN}}{m_{HF@SLN}}\times100\%$

The concentration of HW131 was quantified using HPLC equipped with a UV detector at 254 nm. Separation was performed on a C18 column (150 mm, 4.6 μm) using water (0.1% formic acid) and acetonitrile (0.1% formic acid) as the mobile phases.

The fluorescein (FITC) labeled HF@SLN (HF@SLN-FITC) was prepared by replacing the mPEG-DSPE to the DSPE-PEG-FITC. Similarly, The Cyanine 7 (Cy7) labeled HF@SLN (HF@SLN-Cy7) was prepared by replacing the mPEG-DSPE to the DSPE-PEG-Cy7. Moreover, sorafenib, pexidartinib, and erlotinib were respectively coordinated with Zn^2+^ with the mole ratios (4:1) in the methanol at 60℃ to prepare the sorafenib aMDF, pexidartinib aMDF, and erlotinib aMDF.

Afterwards the crystal structure of HF@SLN, HF, HW, and Zn(NO_3_)_2_ were explored by the diffraction of x-rays (XRD, Bruker D2 Phaser, Germany) from 5-90°with the speed at 1°/min. Thermogravimetric analysis (TGA, TGA 550, USA) was performed at HF and HF@SLN from room temperature to 800℃ with the heating speed at 5℃/min under an air atmosphere. The hydrodynamic diameter, polydispersity index, and zeta-potential of HF, SLN, HF@SLN, sorafenib aMDF, pexidartinib aMDF, and erlotinib aMDF were evaluated by DLS. The morphology of HW, HF, SLN, and HF@SLN was explored by the transmission Electron Microscope (TEM, FEI). The HF@SLN was incubated in DMEM-high glucose medium with 10% FBS or 1×phosphate buffered saline (PBS) for 3 days, the hydrodynamic diameter and polydispersity index of HF@SLN were monitored by DLS. All nanoparticle formulations used in this study were freshly prepared and used immediately for the subsequent biological experiments.

***In vitro* characterization of the NPs**

M2 BMDMs were co-cultured with HF@SLN-FITC for 2 h and 6 h, then the uptake efficacy of HF@SLN#FITC on M2 BMDMs were imaged by the confocal microscopy (LSM900, ZEISS).

M2 BMDMs were co-cultured with 10 µM HW, and HF@SLN for 2 h and 6 h, and M2 BMDMs cultured without any drugs or NPs were regarded as negative control (NC). After different treatments, M2 BMDMs were stained with the LysoTracker staining (LysoTracker Red DND-99, Yeasen, 40739ES50), and the cell nucleus were stained with 4',6-diamidino-2-phenylindole (DAPI), then these M2 BMDMs were imaged by the confocal microscopy (LSM900, ZEISS). Additionally, these M2 BMDMs were also quantitatively analyzed by the flow cytometry.

M2 BMDMs were, respectively, incubated with only medium, 10 µM HW and HF@SLN for 24 h. After 24 h, the suspension was discarded, cells were wash with PBS three times and stained with CD80-PE and CD206-BV785 (Biolegend) for 30 min with the light protection. Then the surficial markers, CD80 and CD206, were quantitatively analyzed by the flow cytometry.

M2 BMDMs were respectively incubated only medium, 10 µM HW and HF@SLN for 24 h. Then M2 BMDMs were washed with 1× PBS and fixed with 4% paraformaldehyde (PFA), followed by permeabilizated with 0.5% triton X-100. Afterward, cells were blocked with 2% bovine serum albumin (BSA) and incubated with anti-transcription factor EB (TFEB) at 4℃ overnight, followed by incubated with secondary anti-body for 30 min at room temperature. Finally, the nucleus was stained with DAPI and the cells were observed by the confocal microscopy.

Additionally, M2 BMDMs were respectively treated with only medium and HF@SLN for 24 h. The expression of TFEB was also quantitatively analyzed by the Western blotting.

***In vivo* biodistribution of NPs**

3×10^6^ Pan02 cells were subcutaneously injected into the right back of mice under the anaesthetization and analgesics. After 10 days, the Cy7-lablled HF@SLN was intravenously injected into the mice. The accumulation of HF@SLN on the tumor was monitored after 0.5-, 2-, 4-, 6-, 8-, and 24-h injection by the in vivo imaging system (IVIS, Lumina III Series). Furthermore, 4 h after injection, mice were euthanized, and the accumulation of HF@SLN in main organs (heart, liver, spleen, lung, kidney, and tumor) were imaged by the IVIS. Five mice were used in this experiment.

**Evaluation of the NPs in a subcutaneous tumor model**

On day -9, the C57BL/6J mice were subcutaneously injected with 3×10^6^ Pan02 cells on the right back under the anaesthetization and analgesics. On day -2, the mice were pre-treated with anaesthetization and analgesics, then part of tumor was surgically removed and the wound was sutured with absorbable suture to establish the Pan02-bearing surgically removed tumor model. Mice were intraperitoneally injected with analgesics for the following days until the wound was healed. On day 0, under the anaesthetization of isoflurane, the tumor was monitored by the IVIS on each mouse. After that, the mice were randomly divided into three groups with different treatments (saline, HW+50mg/kg gemcitabine, and HF@SLN+50mg/kg gemcitabine) every two days, 10 mice for each groups. During the therapeutic process, the body weight was recorded. After 30 days of treatments, the tumor was monitored by IVIS. With the addition of heparin, blood samples were collected and centrifuged at 900×g for 15 min to isolate the serum. The concentration of alanine aminotransferase (ALT), aspartate aminotransferase (AST), transforming growth factor-β (TGF-β), tumor necrosis factor-alpha (TNF-α), and interleukin 10 (IL-10) were quantitatively analyzed by the enzyme-linked immunosorbent assay (ELISA) in serum. Additionally, the heart, liver, spleen, lung, and kidney were processed for the histology analysis by the hematoxylin and eosin (HE) stain.

Next, tumors were isolated and weighted. After that, tumors were equally divided into three parts. One parallel was cut into small pieces and digested in the cocktail RPMI medium (1 mg/ml Collagenase I, 2 U/mL hyaluronidase, and 0.1 mg/ml deoxyribonuclease) for 30 min at 37°C with constant stirring (200 rpm). Afterwards tumor tissues were gently smashed and filtered with a 70 µm cell strainer (BD falcon). Then the suspension was centrifuged to obtain the cell pellet, and the cell pellet was re-suspended with red blood cell lysis buffer for 3 min and centrifuged at 500g for 5 min (4°C) to remove any red blood cells. After the cell pellet was washed with 1× PBS and blocked with CD16/32 (Biolegend) for 15 min at 4°C and stained with Zombie NIR (Biolegend) for 30 min under the light protection at room temperature. Then the cell pellet was equally divided into two samples. One sample was co-stained with the CD45-PE/Cy7, CD11b-BV510, F4/80-Alexa Fluor 647, CD80-PE, and CD206-BV785 (Biolegend), and another one was co-stained with CD3-FITC, CD8-Percp, CD45-PE/Cy7, and CD4-BV610 (Biolegend) for 30 min at 4°C under the light protection. Cells were washed with 1× PBS to remove any unconjugated antibody and analyzed by flow cytometry.

Another parallel was prepared for the transcriptome analysis. Specifically, tumor tissues were homogenized with the addition of 1mL TRLzol, followed by 5 min of incubation at room temperature to dissociate the ribosome. 0.2 ml chloroform was added into the sample with violent shock, followed by 3 min of incubation. Then samples were centrifugated at 12000 rpm for 15 min (4°C), and the upper layer was insolated for the following experiments. The equal volume of isopropanol was incubated with the upper layer solution for 10 mins (room temperature), followed by the 10 min of centrifugation (4°C, 12000 rpm). Afterwards the suspension was discarded, the pellet was washed with 75% ethanol and centrifugated at 12000 rpm for 3 mins (4°C). Finally, the suspension was discarded and the pellet was dissolved in 100 µl RNase free water.

Next, the Oligo (dT) beads were used to concentrate the mRNA. Then these mRNA was break into short segments by the fragmentation buffer. The first complementary DNA (cDNA) chain was synthesised by random hexamers according to the mRNA template, followed by the addition of buffers, dNTPs, RNase, and DNA polymeraseI to prepared the second cDNA chains. The double chains cDNA was purified by the kits. Finally, the polymerase chain reaction (PCR) was applied to amplificated, and the established sequencing library was used for the sequencing.

**Evaluation of the NPs in orthotopic tumor model**

On day -7, 7×10^6^ Pan02 cells were orthotopically injected to the pancreas of each C57BL/6J mice under the anaesthetization and analgesics, and wounds were sutured with the absorbable suture. For the following days, mice were treated with analgesics until the wound was healed.

On day 0, under the anaesthetization of isoflurane, the orthotopic tumor was monitored by the IVIS systems. After that, the mice were randomly divided into three groups with different treatments (saline, HW+50mg/kg gemcitabine, and HF@SLN+50mg/kg gemcitabine) every two days, 10 mice for each groups. During the whole therapeutic process, the orthotopic tumor was monitored on day 14 and day 28 by the IVIS. The body weight and survival rate were recorded during the 30 days. After 30 days, tumor was collected and weighted. Furthermore, the tumor was processed into the single cell suspension as abovementioned descriptions. Then the single cell suspension was divided into two parallel, one parallel was co-stained with the CD45-PE/Cy7, CD11b-BV510, F4/80-Alexa Fluor 647, CD80-PE, and CD206-BV785 (Biolegend), and another one was co-stained with CD3-FITC, CD8-Percp, CD45-PE/Cy7, and CD4-BV610 (Biolegend). Finally, the two parallels were analyzed by flow cytometry.

**Evaluation of the NPs in insolated human tumor tissues**

The pancreatic ductal adenocarcinoma tumor tissues were isolated from patients following resection in the operating room, tumor tissues were isolated from at least 3 different sites of lesion and immersed in tissue storage solution (Miltenyi), followed by transporting with ice immediately. Then these tumor tissues were, respectively, co-cultured with saline, HW, and HF@SLN in the RPMI-1640 for 24 h in the incubator. Afterwards these tissues were immobilized with 4% paraformaldehyde and stored at 4°C, until tissues were processed with the immunofluorescence staining with FITC-labeled TFEB.

**Statistical analysis**

Data were analyzed by SPSS 20 statistical software (version 20.0) using ANOVA with Tukey’s post-test used for statistical analysis. Error bars indicate mean ± standard deviation (SD) for n ≥ 3 independent samples. P-values on graphs are denoted within each figure panel as ^*, #^P < 0.05 (significant), ^**, ##^P < 0.01 (moderately significant), and ^***, ###^P < 0.001 (highly significant).

**Figure. S1.** Chemical structures of the fifteen pyrrolopyrimidine analogs.

**Figure. S2.** Morphology of M2 macrophages without any treatments or with 2 h incubation with 10 µM HW.

**Figure. S3.** Characterization of HF@SLN. (A) TEM images of HW. XRD spectrum of (B) HF, (C) HF@SLN, (D) HW, and (E) SLN.

**Figure. S4.** Characterization of sorafenib, pexidartinib, and erlotinib aMDF in (A) Size, (B) Zeta potentials, and (C) PDI.

**Figure. S5.** H&E staining images of main organs from mice treated with saline, HW, and HF@SLN after 30 days.

**Figure S6. Gating strategies for spleen macrophages**

**Figure S7. Gating strategies for tumor macrophages**

**Figure S8. Gating strategies for spleen T cells**

**Figure S9. Gating strategies for tumor T cells**

**Figure. S10.** Body weight of the orthotopic Pan02-bearing mice treated with saline, HW, and HF@SLN.

**Figure. S11.** TFEB staining images of human pancreatic cancer slice treated with saline. HW, and HF@SLN after 24 h of incubation. The green fluorescence indicated the expression of TFEB, and the blue fluorescence indicate the nucleus.
